# Giant Hepatic Carcinoid: A Rare Tumor with a Favorable Prognosis

**DOI:** 10.1155/2014/456509

**Published:** 2014-02-09

**Authors:** Serge Landen, Maxime Elens, Celine Vrancken, Frederiek Nuytens, Thibault Meert, Veronique Delugeau

**Affiliations:** ^1^Department of Surgery, St Elizabeth Hospital, Avenue De Fré 206, 1180 Brussels, Belgium; ^2^Department of Medicine, Clinique La Ramée, Avenue Boetendael 34, 1180 Brussels, Belgium

## Abstract

Primary hepatic carcinoids are rare tumors that are often diagnosed at a locally advanced stage. Their primary nature can only be ascertained after thorough investigations and long-term follow-up to exclude another primary origin. As with secondary neuroendocrine liver tumors, surgical resection remains the mainstay of therapy. Despite their large size and often central location liver resection is often feasible, offering long-term survival and cure to most patients. In selected patients liver transplantation appears to be a good indication for tumors not amenable to liver resection. An aggressive surgical attitude is therefore warranted. We report a large and unusually fast-growing liver carcinoid that appeared only marginally resectable in a patient who remains free of disease four years after surgery.

## 1. Introduction

With less than 100 reports in the literature little is known of primary hepatic carcinoids. They often present as large centrally situated liver masses, characteristics that may discourage attempts at resection. Data is scarce on the outcome of surgical treatment but these tumors seem to be associated with a favorable prognosis, justifying an aggressive surgical approach. Presented here is a patient with a giant primary liver carcinoid that appeared only marginally resectable, who remains disease free four years after surgical resection.

## 2. Case Report

**Figure 1:**
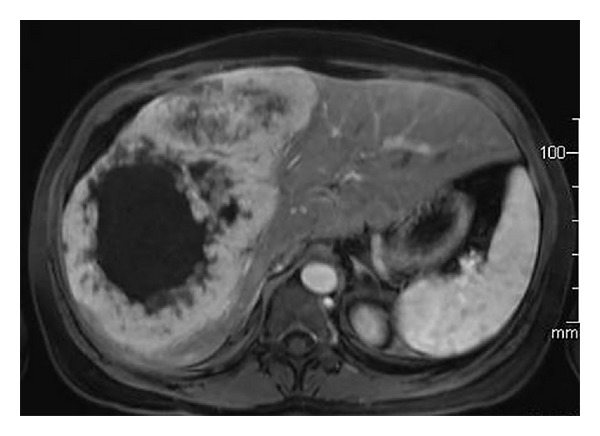
Transverse section of arterial phase of magnetic resonance: large right liver lobe tumor exhibiting early peripheral enhancement and a central cystic component.

A 52-year-old female complained of fatigue and intermittent fever of one-year duration. There was no history of liver failure, hematemesis, flushing, or diarrhea. She had undergone a previous appendectomy. Abdominal ultrasound revealed a heterogeneous 8 cm right lobe focal liver lesion. Contrast enhanced CT performed one month later showed a solid 15 cm liver mass with a hypodense core that was suggestive of an atypical hemangioma. The patient came to our attention two months after the initial ultrasound with palpable hepatomegaly. MR showed a hypervascularized solitary liver lesion measuring 20 cm (Figure 1). Malignancy was further suspected by FDG-PET scan showing increased uptake. Laboratory data showed normal liver function and hepatitis B and C serologies were negative. Serum tumor markers including CEA, AFP, CA 12.5, CA 19.9, and NSE were within normal range while chromogranin A was moderately elevated. 24-hour urinary 5-HIAA excretion was normal. Chest CT showed no signs of malignancy. No primary tumor was found at gastroscopy and colonoscopy. Laparoscopy showed that the right liver lobe was completely occupied by a polylobulated firm white mass. The left lobe appeared healthy and liver biopsy confirmed this. There were no serosal surface tumor deposits and no lymphadenopathy at the porta hepatis. Differential diagnosis included hepatocellular carcinoma, cholangiocellular carcinoma, hypervascularized metastasis, angiosarcoma, hemangiopericytoma, and a neuroendocrine tumor.

During laparotomy thorough exploration of the abdominal cavity, small bowel, and mesentery was performed before proceeding to an extended right hepatectomy. The resected specimen weighed 2.2 kg and was almost entirely occupied by a 22 cm solid mass with a large central cystic component. Microscopically the tumor displayed a trabecular and pseudoglandular pattern and a highly vascular stroma. Tumor cells were uniform and displayed rare mitoses (<1 per mm³). At immunohistochemical examination tumor cells stained positive with chromogranin A and synaptophysin antibodies and were negative for hepatocyte, AFP, and CD56 antibodies. 10% nuclear reactivity for Ki-67 was present. There was intense expression of somatostatin subtypes I and II receptors but not subtype V.

Final diagnosis was a well-differentiated nonsecreting neuroendocrine tumor. Further investigations including In^111^-DTPA-octreotide scan, EUS of the pancreas, thyroid US and Tc-scintigraphy, and small bowel barium study failed to find a primary neuroendocrine tumor. Review of the pathology of the previous appendectomy was also noncontributive. Postoperative screening for recurrence and detection of a possible primary tumor has included abdominal MR, chest X-ray, and serum chromogranin A assay every 6 months. Octreotide and FDG-PET scan are scheduled yearly. At 48-month follow-up the patient shows no signs of liver recurrence or appearance of a primary tumor or secondary extrahepatic tumor. She is asymptomatic and fully functional.

## 3. Discussion

Carcinoid tumors also known as well-differentiated neuroendocrine tumors (NET) derive from neuroectodermal cells that are dispersed throughout the digestive tract but are also found in organs such as the adrenals, bronchi, thymus, thyroid, and paravertebral ganglia. Fifty-four percent of carcinoids occur within the gastrointestinal tract, mostly in the appendix and small bowel (16.7% and 44.7% resp.) but also in the rectum (19.6%), colon (10.6%), and stomach (7.2%) [1]. Carcinoids also occur in the lung (30.1%), pancreas (2.3%), genitals (1.2%), biliary tract (1.1%), and head and neck (0.4%). In the United States the incidence of carcinoid tumors is 6.25 cases per 100000 per year [2].

The 2010 World Health Organization carcinoid and pancreatic neuroendocrine tumor grading system takes into account the number of mitoses per 10 high power microscopic fields or the percentage of tumor cells that immunolabel positively for Ki-67 antigen. These measures reflect the rate of proliferation and correlate with prognosis. Carcinoids are classified into three types: (1) well-differentiated tumors of low grade malignancy with an indolent development and a good prognosis, (2) moderately differentiated or intermediate grade neoplasms, and (3) poorly differentiated or high grade epithelial neoplasms that carry a poor prognosis (Table 1).

Less than 100 patients with primary hepatic carcinoid tumors (PHCT) have been reported, mostly as single cases [3]. The two largest series comprise 11 and 8 patients [4, 5]. The tumor occurs mostly in middle age (mean 49.8 years) with a slight female predominance (58.5%). Carcinoid tumors typically grow slowly and become clinically evident only at an advanced stage. Symptoms include abdominal pain (44%), abdominal mass (14.3%), and fatigue (7.1%). Carcinoid syndrome, characterized by flushing, abdominal pain, diarrhea, wheezing, and right heart failure is present in a mere 16.7% of patients. Cushing and Zollinger-Ellison syndromes are present in 2.4% and 6% of patients, respectively. Hence most PHCTs are nonsecreting although few studies actually assessed the secretion of serotonin, histamine, bradykinin, gastrin, vasoactive intestinal peptide, insulin, glucagon, or prostaglandins in the systemic circulation. When reported, the most frequently secreted hormones were gastrin (10.1%) and chromogranin A (7.2%) [6].

Ascertaining that a carcinoid liver tumor is a primary rather than a secondary deposit is challenging. A single large centrally situated tumor is suggestive of a primary tumor whereas neuroendocrine liver metastases present typically as multiple diffuse liver masses [5]. The pancreas is the most common primary site (35%) of neuroendocrine liver metastases [7]. However in 11–14% of patients with liver carcinoids no primary tumor is found. Thorough pre- and intraoperative investigations are required before concluding to a primary liver carcinoid [8]. These include computerized tomography, magnetic resonance, CT or MR enteroclysis, somatostatin scintigraphy, PET scan, gastroscopy, colonoscopy, endoscopic ultrasound of the pancreas, bronchoscopy, video capsule endoscopy or balloon enteroscopy, and operative exploration. In patients having previously undergone appendectomy the pathology report should be reviewed to exclude a primary tumor. Even when after thorough investigation a primary tumor is not identified, long-term reevaluation with conventional imaging, octreotide scan, and possibly PET scan is useful to detect a small primary tumor that may have initially been overlooked.

The best practice for neuroendocrine tumors metastatic to the liver remains surgical resection of both the primary tumor and liver metastases whenever possible. Recent publications indicate an advantage for aggressive liver surgery for locally advanced and metastatic NET in terms of duration and quality of life [9]. Surgical resection even seems to benefit patients with positive resection margins [10]. A Mayo Clinic study on secondary liver NET showed no difference in survival between patients having complete resection and those having 90% resection of their liver secondaries. Supported by a 75% four-year survival rate the authors considered resection to be indicated if the primary tumor and at least 90% of the metastatic tumor burden to the liver could be resected or ablated [11]. Reports indicate 5-year survival rates in the range of 47–92% after resection of NET liver metastases. This contrasts with a 20–30% 5-year survival in historical controls having not undergone liver resection [12]. However recurrences, mainly in the liver, remain high (78%–84%), occurring after a median of 19 months [13, 14]. More recently liver transplantation (LT) has been proposed in selected patients that were not amenable to partial liver resection. Initial results were disappointing owing to the lack of patient selection. Noncarcinoid tumors, nongastrointestinal carcinoids, high-grade tumors, or tumors not drained by the portal vein are considered to be associated with worse outcomes [15, 16]. A retrospective analysis of the United Network for Organ Sharing database on LT performed in the US between 1988 and 2008 included 150 patients with metastatic NET who had an overall 1-, 3- and 5-year survival rate of 81%, 65%, and 49%, respectively [17]. Carcinoid and noncarcinoid tumors had similar prognoses. LT for NET afforded similar survival as LT for hepatocellular carcinoma (HCC), a well-established and accepted treatment of HCC. Tumor recurrences for metastatic NET were 31% which is higher than tumor recurrence for HCC (10–15%).

Because there are only sporadic reports, there are currently no established standards for the treatment of PHCT. Primary hepatic carcinoids are associated with a resectability rate of 70%, a 74–78% 5-year survival rate, and an 18% 5-year recurrence rate [18, 19]. Better long-term disease free survival can therefore be expected after resection of PHCT than after resection of NETs of other primary origins. Postoperative intra-abdominal fluid collections and liver related complications (insufficiency or portal vein thrombosis) did have a negative impact on overall survival in one series [5]. Data are even scarcer concerning LT for PHCT. Five patients have been reported [5, 20–22] including 2 males and 3 females ranging from 35 to 50 years of age. Four patients are alive and disease free after 38, 45, 95, and 120 months. One patient had a liver and mesentery recurrence after 54 months. Based on this small data set it appears that PHCT treated by LT could have a better prognosis than liver NET metastases treated by LT [5, 20].

In patients with unresectable disease a variety of palliative options exist but data on these is very limited. Systemic 5-fluorouracil downstaged disease in 1 of 3 patients [23]. Hepatic artery embolization may also be effective because hepatic carcinoids derive their vascular supply from the hepatic artery [24]. Octreotide, a somatostatin analog, can effectively alleviate symptoms resulting from hormone secretion but may also have a direct antiproliferative effect [25]. Yttrium-90 targeted radionuclides coupled with octreotide have also shown some therapeutic effect [26].

In conclusion primary hepatic carcinoid tumors are rare and their primary nature can only be ascertained after thorough investigations and long-term follow-up to exclude another primary origin. Their large size and often central situation within the liver should not deter surgeons from attempting resection because long-term survival and cure can be expected. In selected patients not amenable to partial liver resection liver transplantation can be considered.

## Figures and Tables

**Table 1:** Histopathological classification of neuroendocrine tumors.

Histological classification	Well-differentiated (low grade, G1)	Moderately differentiated (intermediate grade, G2)	Poorly differentiated (high grade, G3)
Appearance	Monomorphic population of small round cells	Undefined	Cellular pleomorphism
Prognosis	Prolonged survival	Intermediate	Poor
Mitotic rate*	<2	2–20	>20
Ki-67 index**	<3%	3–20%	>20%
Necrosis	Absent	Undefined	Present

*Per 2 mm^2^; **percentage of tumor cells that immunolabel positively for Ki-67 antigen.
